# Supplementary material for: Effect of the Landscape on Insect Pests and Associated Natural Enemies in Greenhouses Crops: The Strawberry Study Case
Source: Insects. 2023 Mar 21;14(3):302. doi: 10.3390/insects14030302 (PMC10051428; doi:10.3390/insects14030302)
Supplement: Supplementary file 1 [file insects-14-00302-s001.zip › insects-2256854-supplementary.pdf]

Supplementary Materials:

Supplementary Table S1. Response and variables analyzed in the three published studies on the effect of surrounding landscape on insects in greenhouses.

Response variable	Landscape factors	Buffer size around the greenhouses (m)	Effect of landscape factor	Crop	References
Abundance of mirid predator *Macrolophus spp.*	% Cover of fallow	100 & 300	Increased	Tomato	Aviron *et al.*, 2016
	Connectivity of fallow	100	Increased		
	% cover of grassy orchards	200 & 300	Decreased		
	Connectivity of orchards	200 & 300	Decreased		
Abundance of mirid predator *Dicyphus spp.*	% Cover of grassy orchards	100	Decreased		
Vulnerability[1]	% woodland	500	Decreased	Watermelon	Dong *et al.*, 2019
Active primary parasitism	% woodland	1000	Increased		
Hyperparasitoid richness	% woodland	500	Decreased		
Primary parasitoid richness	% vegetable land[2]	1000	Decreased		
Aphid mortality	% vegetable, %orchard, % grassland, % woodland, % water, and % urban	500, 1000, 2000 & 3000	Not significant		
Early colonisation of mirid predator *Macrolophus pygmaeus*[3]	% herbaceous semi-natural cover	250	Increased[3]	Tomato	Ardanuy *et al.*, 2022

1 : Mean number of hyperparasitoid per primary parasitoid species ; 2: Vegetable contained protected fields, e.g. greenhouses in which vegetable were usually planted; 3 : In interaction with the the presence of *Calendula* banker plants inside the greenhouses.

Supplementary Table S2. Description of the 32 monitored greenhouse strawberry crops.

Greenhouse ID	Department	Greenhouse openess degree	Number of plants sampled at 1st session	Number of plants sampled at 2nd session
1	24	Closed	28	26
2	47	Closed	30	30
3	47	Closed	30	30
4	47	Open	27	21
5	33	Closed	48	25
6	47	Closed	48	30
7	47	Closed	32	Not sampled
8	47	Open	Not sampled	25
9	47	Open	50	34
10	47	Closed	21	21
11	24	Open	21	Not sampled
12	24	Open	Not sampled	21
13	47	Open	45	30
14	47	Open	21	30
15	47	Open	41	40
16	47	Open	27	40
17	47	Open	35	40
18	47	Open	30	Not sampled
19	47	Closed	Not sampled	30
20	24	Closed	24	30
21	47	Open	27	30
22	47	Open	48	30
23	47	Closed	41	30
24	47	Closed	30	30
25	47	Closed	40	30
26	47	Open	34	26
27	47	Closed	21	Not sampled
28	47	Closed	Not sampled	30
29	47	Closed	40	30
30	47	Closed	22	31
31	47	Open	40	34
32	47	Closed	20	25

Supplementary Table S3: Pest management practices used by growers in the 32 monitored greenhouses.

	Release of aphid predators	Release of aphid parasitoids	Release of thrips predators	Use of insecticides
Yes	7	9	31	30
No	25	23	1	2

Supplementary Table S4: Variance Inflation Factors (VIFs) between covariates considered in the statistical modelling.

	VIF values		
**Variables**	**run1**	**run2**	**run3**
% Cereal crop	10.116	**7.097**	Excluded
% Oleaginous crop	4.998	2.929	2.043
% Vegetable crop	1.671	1.666	1.593
% Orchard	6.338	4.917	2.525
% Semi-natural habitat and grassland	5.478	5.420	2.039
% Woodland	5.718	5.015	1.931
% Urban	5.286	2.760	2.512
% Bare ground	3.320	3.151	2.387
% Water	4.056	2.704	2.641
Shannon diversity index	2.974	2.856	2.366
Number of land cover types	2.768	2.331	2.130
Number of patches	46.648	3.058	2.876
Mean patch surface	**37.408**	Excluded	
log (Length of hedges)	4.115	3.232	2.691
